# Supplementary material for: A versatile, fast and unbiased method for estimation of gene-by-environment interaction effects on biobank-scale datasets
Source: Nat Commun. 2023 Aug 25;14:5196. doi: 10.1038/s41467-023-40913-7 (PMC10457310; doi:10.1038/s41467-023-40913-7)
Supplement: Supplementary file 1 — Supplementary Information [file 41467_2023_40913_MOESM1_ESM.pdf]

Supplementary Information

A versatile, fast and unbiased method for estimation of gene-by-environment interaction effects on biobank-scale datasets

**Matteo Di Scipio[1,2], **Mohammad Khan[1,2], Shihong Mao[1], Michael Chong[1,3,4], Conor Judge[1], Nazia Pathan[1,2], Nicolas Perrot[1], Walter Nelson[5,6], Ricky Lali[1,7], Shuang Di[5,8], Robert Morton[1,4], Jeremy Petch[1,2,5,9], *Guillaume Paré[1,3,4,7]

** Contributed equally.

* Corresponding author.

[1]Population Health Research Institute, David Braley Cardiac, Vascular and Stroke Research Institute, Hamilton Health Sciences and McMaster University, Hamilton, Canada. [2]Department of Medicine, Faculty of Health Sciences, McMaster University, Hamilton, ON, Canada. [3]Thrombosis and Atherosclerosis Research Institute, David Braley Cardiac, Vascular and Stroke Research Institute, Hamilton, Canada. [4]Department of Pathology and Molecular Medicine, McMaster University, Michael G. DeGroote School of Medicine, Hamilton, Canada. [5]Centre for Data Science and Digital Health, Hamilton Health Sciences, Hamilton, ON, Canada. [6]Department of Statistical Sciences, University of Toronto, Toronto, ON, Canada. [7]Department of Health Research Methods, Evidence, and Impact, McMaster University, Hamilton, Canada. [8]Dalla Lana School of Public Health, University of Toronto, Toronto, ON, Canada. [9]Institute of Health Policy, Management and Evaluation, University of Toronto, Toronto, ON, Canada.

Supplemental Tables: 8
Supplemental Figures: 6

Corresponding Author:

Guillaume Paré MD, MSc, FRCPC
McMaster University Population Health Research Institute
David Braley Cardiac, Vascular, and Stroke Research Institute

237 Barton Street East – C4 126
Phone: 905-527-4322 x40365; Fax 905-297-3789
E-mail: pareg@mcmaster.ca

Contents

Supplementary Table 1: Calculated Versus Observed Confidence Intervals 1

Supplementary Table 2: Simulations Stratified by MAF and LD 2

Supplementary Table 3: Dichotomous Outcomes Simulations..3

Supplementary Table 4: Imputation Scenarios Simulations 4

Supplementary Table 5: MonsterLM Heritability Estimates without GxE Algorithm Adjustments 5

Supplementary Table 6: Pearson Correlation Coefficients between Marginal Genetic, Interaction, and Exposure Heritability Combinations...6

Supplementary Table 7: MonsterLM Benchmarking Simulations with mtg2 7

Supplementary Table 8: MonsterLM Matrix Inversion Speed...8

Supplementary Figure 1: MonsterLM Power Calculations 9

Supplementary Figure 2: Number of SNPs Included in the Directionality Analysis 10

Supplementary Figure 3: Real-data Analysis Stratified by MAF and LD 11

Supplementary Figure 4: Proportion of Recovered Variance Explained as function of Marginal Genetic and Interaction Univariate p-value Thresholds...12

Supplementary Figure 5: Number of SNPs Included in the Univariate Recovery Analysis 13

Supplementary Figure 6: Polygenic Scores with GxE for C-Reactive Protein 14

Supplementary Table 1. Predicted Versus Observed Variance Estimates to Compute MonsterLM Confidence Intervals.

Scenario	Predicted Variances Average ($\bar{\sigma}^2_{pred.}$) and Observed Variances ($\sigma^2_{G_{obs.}}$) for Heritability (*G*) and GxE (*G*x*E*) Simulation Estimates	
	G: ($\bar{\sigma}^2_{G_{pred.}}, \sigma^2_{G_{obs.}}$)	***G*x*E***: ($\bar{\sigma}^2_{GxE_{pred.}}, \sigma^2_{GxE_{obs.}}$)
1	2.16×10^{-5} , 2.81×10^{-5}	1.94×10^{-5} , 1.06×10^{-5}
2	2.17×10^{-5} , 3.05×10^{-5}	2.04×10^{-5} , 1.44×10^{-5}
3	1.97×10^{-5} , 2.53×10^{-5}	1.84×10^{-5} , 1.14×10^{-5}
4	2.17×10^{-5} , 3.37×10^{-5}	2.04×10^{-5} , 2.51×10^{-5}
5	2.16×10^{-5} , 1.39×10^{-5}	2.04×10^{-5} , 3.16×10^{-5}
6	2.16×10^{-5} , 4.40×10^{-5}	2.04×10^{-5} , 9.24×10^{-6}
7	2.16×10^{-5} , 3.22×10^{-5}	2.05×10^{-5} , 4.39×10^{-6}
8	2.16×10^{-5} , 2.65×10^{-5}	2.06×10^{-5} , 3.53×10^{-5}
9	2.03×10^{-5} , 2.03×10^{-5}	1.82×10^{-5} , 1.58×10^{-5}
10	2.05×10^{-5} , 2.19×10^{-5}	1.61×10^{-5} , 1.05×10^{-5}
11	2.34×10^{-5} , 2.82×10^{-5}	1.61×10^{-5} , 2.56×10^{-5}
12	2.04×10^{-5} , 1.32×10^{-5}	2.05×10^{-5} , 1.07×10^{-5}
Statistical Significance	$p = 0.061$	$p = 0.460$

Precision Calibration. MonsterLM precision concordance between the average predicted variances of the simulated scenarios compared to their observed variance as per the MonsterLM method. Predicted variances averages ($\bar{\sigma}^2_{pred.}$) and observed variances ($\sigma^2_{obs.}$) for heritability (*G*) and interaction (*G*x*E*) simulation estimates are compared. Two sample t-tests are used to assess significant differences between the groups.

Supplementary Table 2. The Average of 10 MonsterLM Scenario 2 Simulation Estimates Stratified by MAF and LD.

Group	SNP Count, *P*	LD Strata	MAF Strata	Average Estimate Per SNP $R^2_{G,i}$	Average Estimate Per SNP $R^2_{GxE,i}$
1	129335	(0,0.25]	(0.05,0.10)	6.318×10^{-7}	2.481×10^{-7}
2	66427	(0,0.25]	(0.10,0.20]	7.580×10^{-7}	3.148×10^{-7}
3	28007	(0,0.25]	(0.20,0.30]	8.496×10^{-7}	3.727×10^{-7}
4	18256	(0,0.25]	(0.30,0.40]	8.845×10^{-7}	3.560×10^{-7}
5	15622	(0,0.25]	(0.40,0.50]	8.656×10^{-7}	3.537×10^{-7}
6	66442	(0.25,0.50]	(0.05,0.10)	9.068×10^{-7}	3.516×10^{-7}
7	79133	(0.25,0.50]	(0.10,0.20]	9.554×10^{-7}	3.703×10^{-7}
8	46717	(0.25,0.50]	(0.20,0.30]	1.133×10^{-6}	4.579×10^{-7}
9	34669	(0.25,0.50]	(0.30,0.40]	1.187×10^{-6}	4.875×10^{-7}
10	30686	(0.25,0.50]	(0.40,0.50]	1.146×10^{-6}	4.509×10^{-7}
11	33574	(0.50,0.75]	(0.05,0.10)	1.091×10^{-6}	4.199×10^{-7}
12	69734	(0.50,0.75]	(0.10,0.20]	1.061×10^{-6}	4.108×10^{-7}
13	57918	(0.50,0.75]	(0.20,0.30]	1.242×10^{-6}	4.853×10^{-7}
14	49730	(0.50,0.75]	(0.30,0.40]	1.296×10^{-6}	5.157×10^{-7}
15	46691	(0.50,0.75]	(0.40,0.50]	1.226×10^{-6}	4.840×10^{-7}
16	12840	(0.75,0.90]	(0.05,0.10)	1.187×10^{-6}	4.907×10^{-7}
17	48347	(0.75,0.90]	(0.10,0.20]	1.018×10^{-6}	4.047×10^{-7}
18	61388	(0.75,0.90]	(0.20,0.30]	1.111×10^{-6}	4.333×10^{-7}
19	65608	(0.75,0.90]	(0.30,0.40]	1.143×10^{-6}	4.483×10^{-7}
20	69464	(0.75,0.90]	(0.40,0.50]	1.057×10^{-6}	3.9292×10^{-7}

MAF/LD Stratification Simulations. The average estimate across 10 simulations for each MAF/LD strata. Each stratum estimate is divided by the number of SNPs included. Scenario 2 is applied and $R^2_{G,i}$ and $R^2_{GxE,i}$ represent the per SNP average for heritability and *G*x*E* estimates for each group, respectively. Non-zero effect SNPs are randomly distributed across all strata.

Supplementary Table 3. Dichotomous Outcomes for Three Simulations.

Model (R^2_{set})	Simulation 1: Dichotomous Outcome R^2 (CI)	Simulation 2: Dichotomous Outcome R^2 (CI)	Simulation 3: Dichotomous Outcome R^2 (CI)
G (0.20)	0.119 (0.024 , 0.214)	0.203 (0.108 , 0.299)	0.194 (0.099 , 0.0289)
*G*x*E* (0.00)	0.018 (-0.143 , 0.179)	0.089 (-0.073 , 0.250)	0.045 (-0.116 , 0.206)

Dichotomous Outcome Simulations. MonsterLM *G* and *G*x*E* simulated genome-wide three times to assess dichotomous outcome estimation accuracy.

Supplementary Table 4. MonsterLM Performance with Varying Imputation Conditions.

**Model (R^2_{set})**	**20% *Y* Imputation Average, $\overline{R^2}$ (σ)**	**20% *E* Imputation Average, $\overline{R^2}$ (σ)**	**20% *E, Y* Imputation Average, $\overline{R^2}$ (σ)**	**0% *E, Y* Imputation Average, $\overline{R^2}$ (σ)**
G (0.20)	0.1660 (0.0046)	0.2071 (0.0047)	0.1660 (0.0046)	0.2054 (0.0047)
*G*x*E* (0.00)	0.0717 (0.0045)	0.0660 (0.0045)	0.8969 (0.0054)	0.0908 (0.0045)

Imputation Scenarios. MonsterLM performance using ten base model genome-wide (scenario 2) simulations with specific imputation conditions. Average heritability estimates (*G*) compared to the set point of 0.20 and *G*x*E* estimates compared to the set point of 0.10 under the following four imputation conditions: i) 20% of the outcome (*Y*) is mean imputed, ii) 20% of the exposure (*E*) is mean imputed, iii) 20% of the same *E* and *Y* individuals are mean imputed, and iv) no *E* or *Y* mean imputation of the exposure (*E*) is mean imputed, iii) 20% of the same *E* and *Y* individuals are mean imputed, and iv) no *E* or *Y* mean imputation.

Supplementary Table 5. MonsterLM Additive Genetic Variance (Heritability, h_G^2) without Exposure Adjustments.

Trait	MonsterLM h_G^2 (95% CI)
Apolipoprotein A	0.304 (0.293 , 0.314)
Apolipoprotein B	0.220 (0.210 , 0.229)
Cholesterol	0.178 (0.169 , 0.187)
CRP	0.280 (0.271 , 0.290)
Glucose	0.112 (0.102 , 0.122)
HDL-Cholesterol	0.364 (0.354 , 0.375)
HbA1c	0.309 (0.299 , 0.319)
Height	0.691 (0.680 , 0.701)
LDL-Cholesterol	0.171 (0.162 , 0.181)
Triglycerides	0.257 (0.247 , 0.266)
Total Bilirubin	0.404 (0.394 , 0.414)
Waist-Hip Ratio	0.226 (0.217 , 0.235)

MonsterLM Heritability Estimates. MonsterLM heritability estimates applied without the exposure adjustments of residualization and heteroskedasticity normalization described in the methods.

Supplementary Table 6. MonsterLM Pearson Correlation Coefficients with Marginal Genetic, Interaction, and WHR Heritability Effects.

**Trait**	**Corr($\hat{\beta}_G, \hat{\beta}_{h^2_{WHR}}$)**	**Corr($\hat{\beta}_{GE}, \hat{\beta}_{h^2_{WHR}}$)**	**Corr($\hat{\beta}_G, \hat{\beta}_{GE}$)**
Apolipoprotein A	-0.00266	-0.00658	0.028173*
Apolipoprotein B	-0.01722*	-0.00946	0.050714*
Cholesterol	-0.01693*	-0.01242	0.072548*
CRP	0.000326	0.001379	-0.0372*
Glucose	0.007645	-0.00164	0.049059*
HbA1c	0.008335	0.000121	0.062878*
HDL-Cholesterol	-0.00348	-0.00514	0.038071*
LDL-Cholesterol	-0.01741*	-0.01281*	0.055702*
Triglycerides	9.76x10-5	-0.00653	0.015742*
Height	0.009866	0.002599	0.015919*
Total Bilirubin	-0.01009	0.00417	0.006513

Pearson Correlation Coefficients Genetic, Interaction, and WHR (exposure) heritability regression coefficients. For each outcome calculated in MonsterLM, a Pearson correlation test is performed between each pair of regression coefficients ($\hat{\beta}_{1xm}$) in the three combinations above. $\hat{\beta}_G$, $\hat{\beta}_{GE}$, $\hat{\beta}_{h^2_{WHR}}$: Total genetic regression coefficients, total interaction regression coefficients, and total WHR heritability regression coefficients, respectively. Significant Pearson correlation tests at *p<0.05/11.

Supplementary Table 7. MonsterLM Simulation Benchmarking with mtg2 GxE.

**Simulation ($R^2_{GW\_set}$, $R^2_{GWEI\_set}$)**	**MonsterLM $R^2_{GW}(\sigma)$**	**mtg2 $R^2_{GW}(\sigma)$**	**MonsterLM $R^2_{GWEI}(\sigma)$**	**mtg2 $R^2_{GWEI}(\sigma)$**
1 (0.2, 0.1)	0.1999 (0.0045)	0.2161 (0.0067)	0.0895 (0.0080)	0.1026 (0.0042)
2 (0.2, 0.1)	0.2038 (0.0045)	0.1251 (0.0039)	0.0949 (0.0080)	0.1249 (0.0034)
3 (0.2, 0.1)	0.2029 (0.0045)	0.2191 (0.0067)	0.0958 (0.0080)	0.1000 (0.0041)
4 (0.2, 0.1)	0.2084 (0.0045)	0.2241 (0.0067)	0.0881 (0.0080)	0.0951 (0.0041)
5 (0.2, 0.1)	0.2080 (0.0045)	0.2339 (0.0068)	0.0887 (0.0080)	0.0994 (0.0041)
6 (0.2, 0.0)	0.1965 (0.0045)	0.2291 (0.0069)	-0.0029 (0.0079)	-0.003 (0.0028)
7 (0.2, 0.0)	0.2081 (0.0045)	0.2286 (0.0069)	0.0003 (0.0080)	0.0025 (0.0029)
8 (0.2, 0.0)	0.2004 (0.0045)	0.2339 (0.0069)	0.0041 (0.0080)	0.0006 (0.0029)
9 (0.2, 0.0)	0.2083 (0.0045)	0.125 (0.0038)	-0.0039 (0.0080)	0.125 (0.0038)
10 (0.2, 0.0)	0.2111 (0.0045)	0.125 (0.0038)	0.0024 (0.0080)	0.125 (0.0038)

Simulation Benchmarking. MonsterLM and mtg2 G and GxE methods are compared genome-wide using the same simulated phenotypes and exposures with set points described in $R^2_{GWE\_set}$. Total estimates for each model are displayed as $R^2_{GW\ or\ GWEI}(\sigma)$ with their respective calculated standard deviation. MonsterLM was calculated with a participant list of N=325,989 and mtg2 was calculated with a participant list of N=75,000.

Supplementary Table 8. MonsterLM Matrix Inversion Speed by Regressor Dimensions with GPULS Conjugate Gradient Method to Least Squares Inversion for Beta.

# of Features	Inversion Time (h)	CG Time (h)	Inversion MSE for Beta	CG MSE for Beta
1,000	0.033	0.014	0.0001	0.0001
2,000	0.11	0.013	5.63×10^{-5}	5.62×10^{-5}
4,000	0.526	0.023	2.88×10^{-5}	2.88×10^{-5}
8,000	3.53	0.065	1.38×10^{-5}	1.38×10^{-5}
12,000	10.364	0.129	9.12×10^{-6}	9.12×10^{-6}
16,000	22.208	0.228	6.93×10^{-6}	6.93×10^{-6}
24,000	69.41	0.496	4.65×10^{-6}	4.65×10^{-6}
32,000	169.099	0.904	3.47×10^{-6}	3.47×10^{-6}

Matrix Inversion Speed Gains. A speed comparison with side-by-side estimates illustrating the length of time to compute each genotype or *G*x*E* regressor by matrix dimensions. CG: conjugate gradient; MSE: mean squared error.

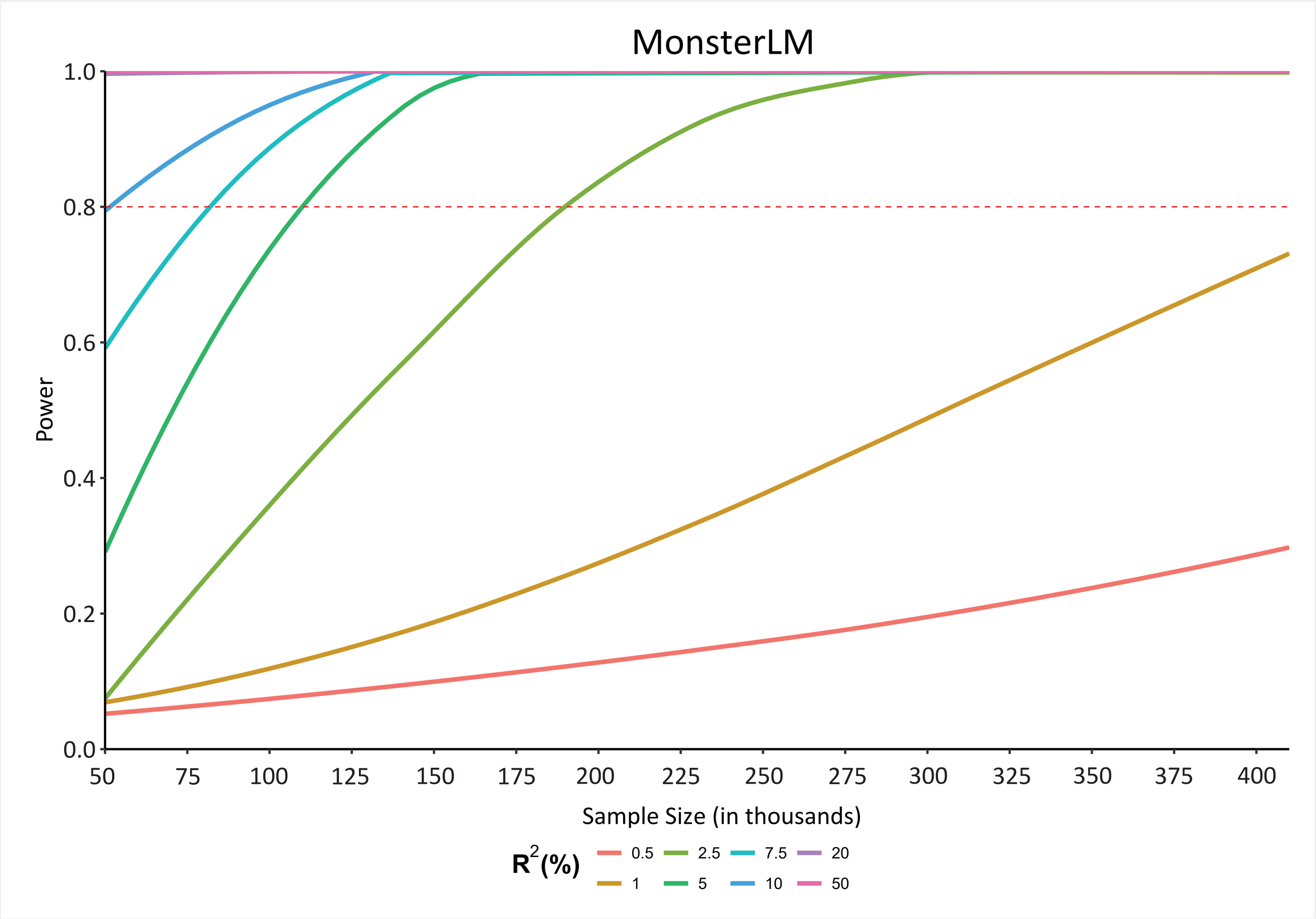

Supplementary Figure 1. MonsterLM power calculations. Power estimates were simulated 10,000 times at every 10,000 sample size interval. Power with varying true set coefficients of determination and sample sizes is displayed. Dashed red line indicates 80% power threshold. Source data are provided as a Source Data file.

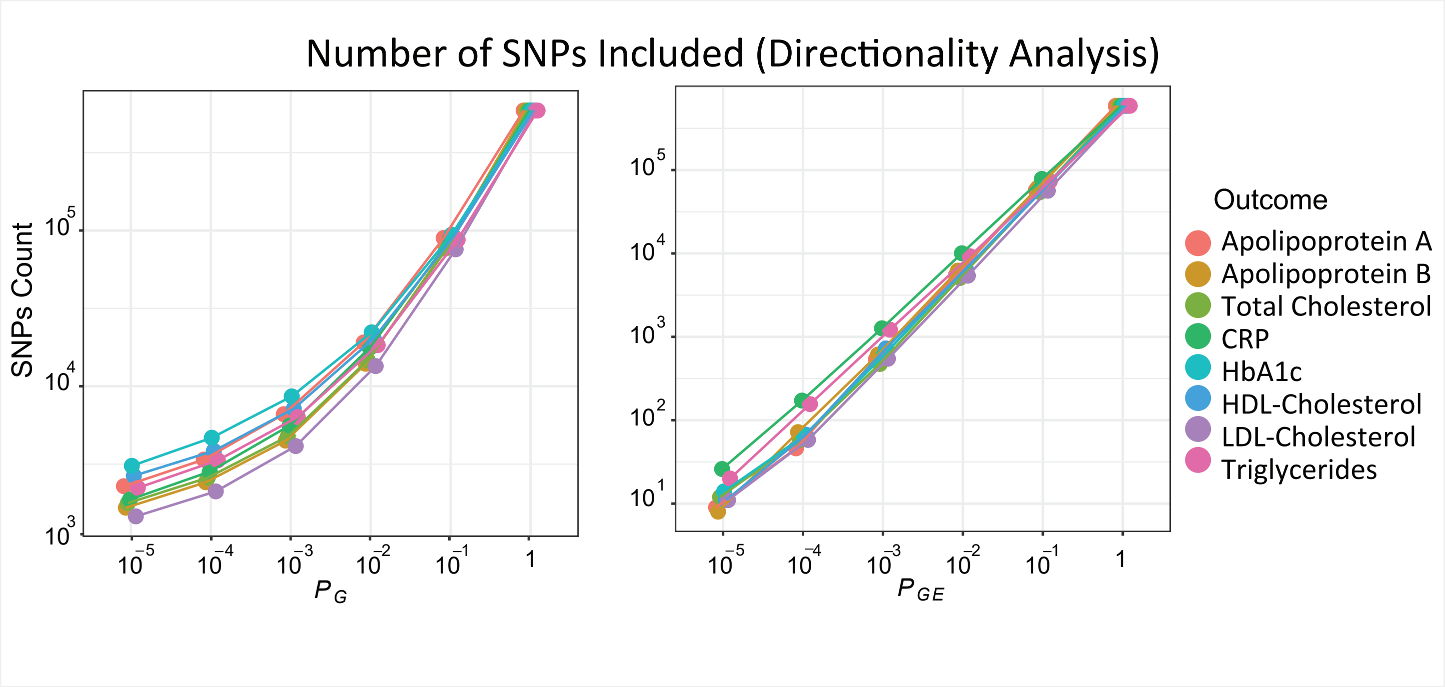

Supplementary Figure 2. Number of SNPs included in the directionality analysis by P_G and P_{GE} thresholds for each outcome. Source data are provided as a Source Data file.

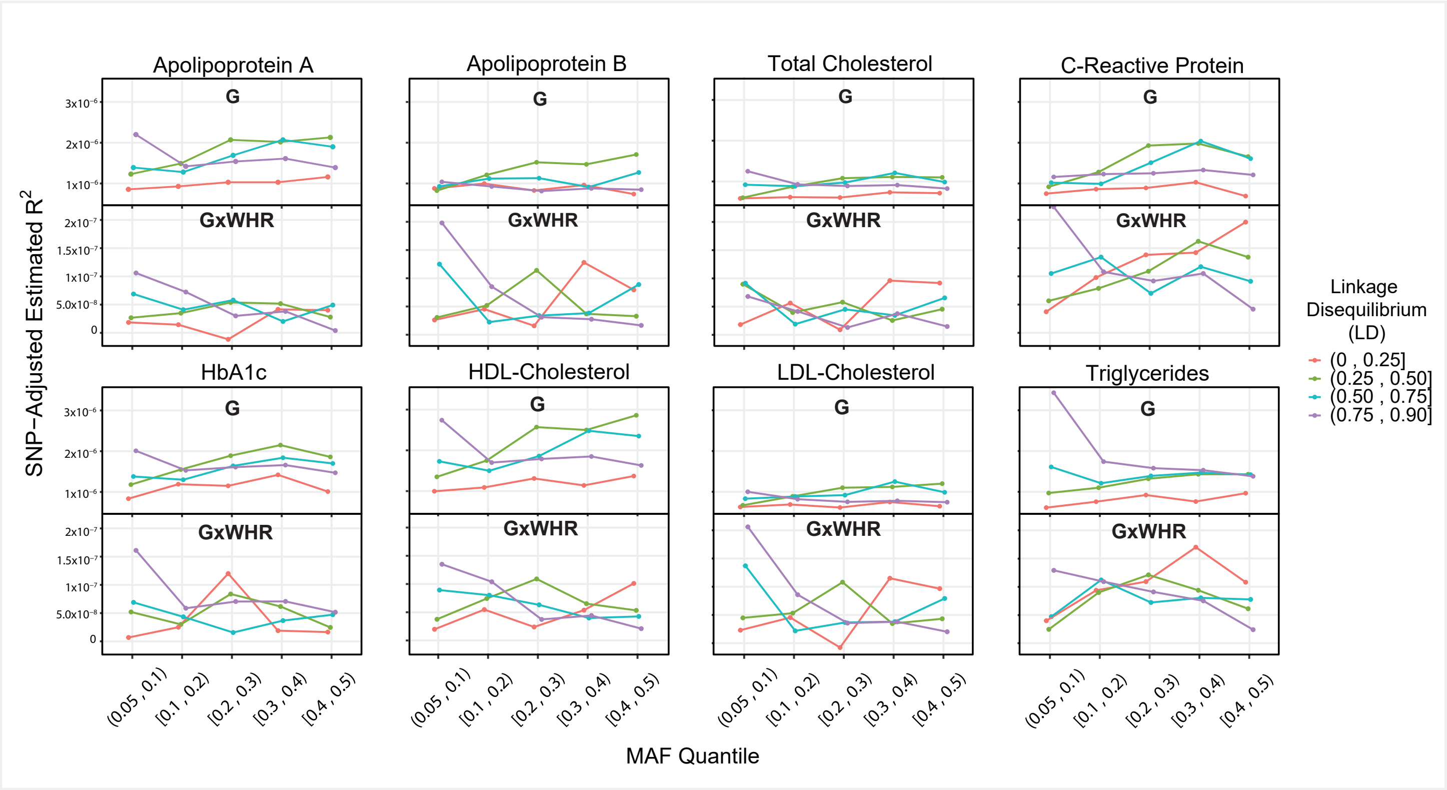

Supplementary Figure 3. Results for WHR are stratified by MAF and LD conditions. Each significant interaction outcome has a per SNP estimate for one of twenty stratified MAF and LD conditions. The top panels (G) per outcome is the heritability stratified estimate per SNP and the bottom panels (GxWHR) per biomarker is the GxE_{WHR} stratified estimate per SNP. SNP-adjusted R^2 is the adjusted R^2 divided by the number of SNPs per stratum. Source data are provided as a Source Data file.

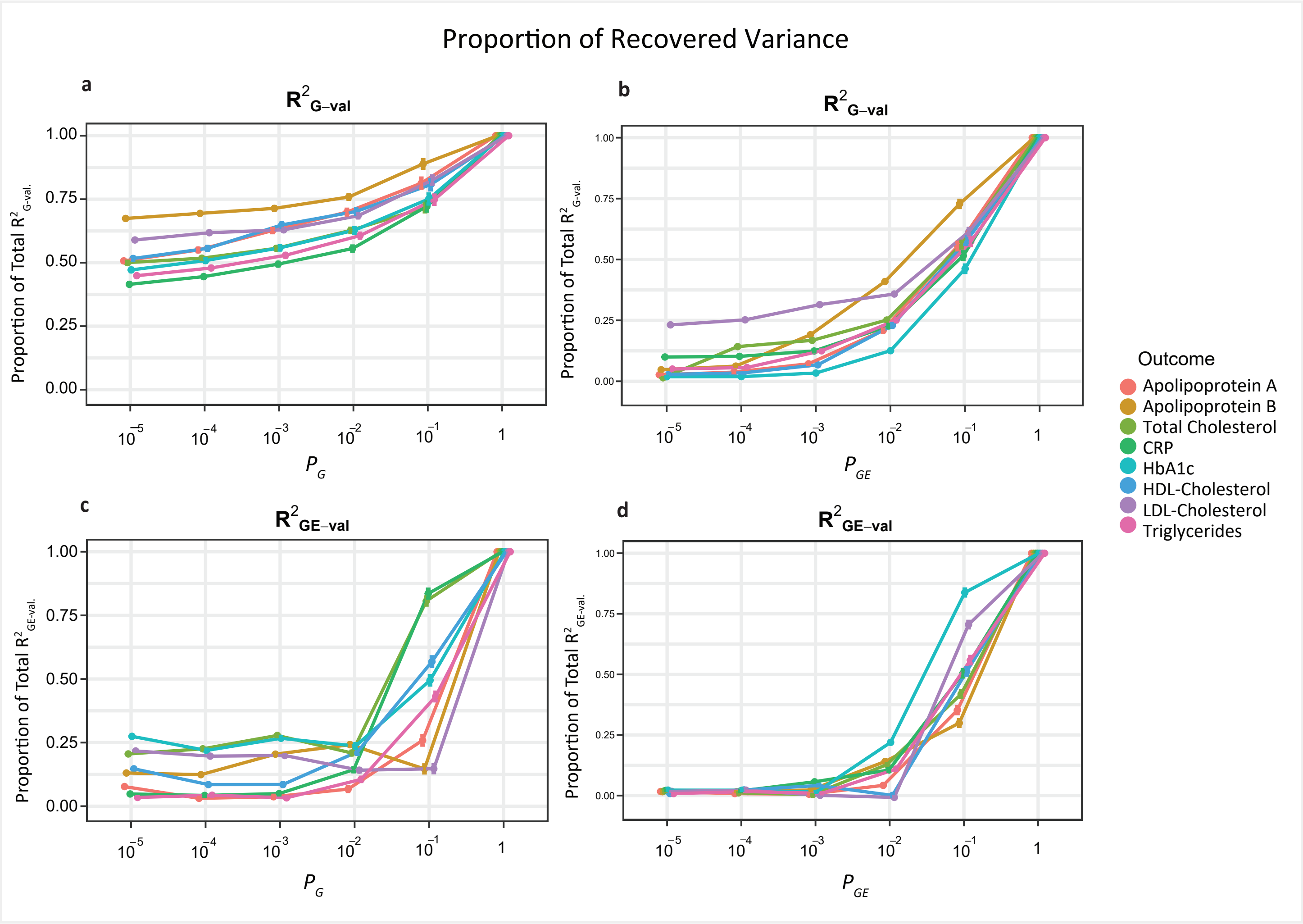

Supplementary Figure 4. Proportion of $R^2_{G\text{-}val.}$ and $R^2_{GE\text{-}val.}$ as a function of P_G and P_{GE}. **a**, The proportion of total $R^2_{G\text{-}val.}$ recovered in the validation set at each discovery sample P_G for the eight outcomes with significant interaction variance; **b**, and for P_{GE} thresholds. **c**, The proportion of total interaction $R^2_{GE\text{-}val}$ recovered in the validation set at each discovery sample P_G threshold for the same outcomes; **d**, and for P_{GE} thresholds. 95% CI were derived based on the upper and lower bounds of each estimate in proportion to either total $R^2_{G\text{-}val.}$ or $R^2_{GE\text{-}val.}$. Estimates were conducted with 325,989 individuals and 1,030,579 SNPs after quality control. Dot and whiskers represent estimates and 95% confidence intervals respectively. Source data are provided as a Source Data file.

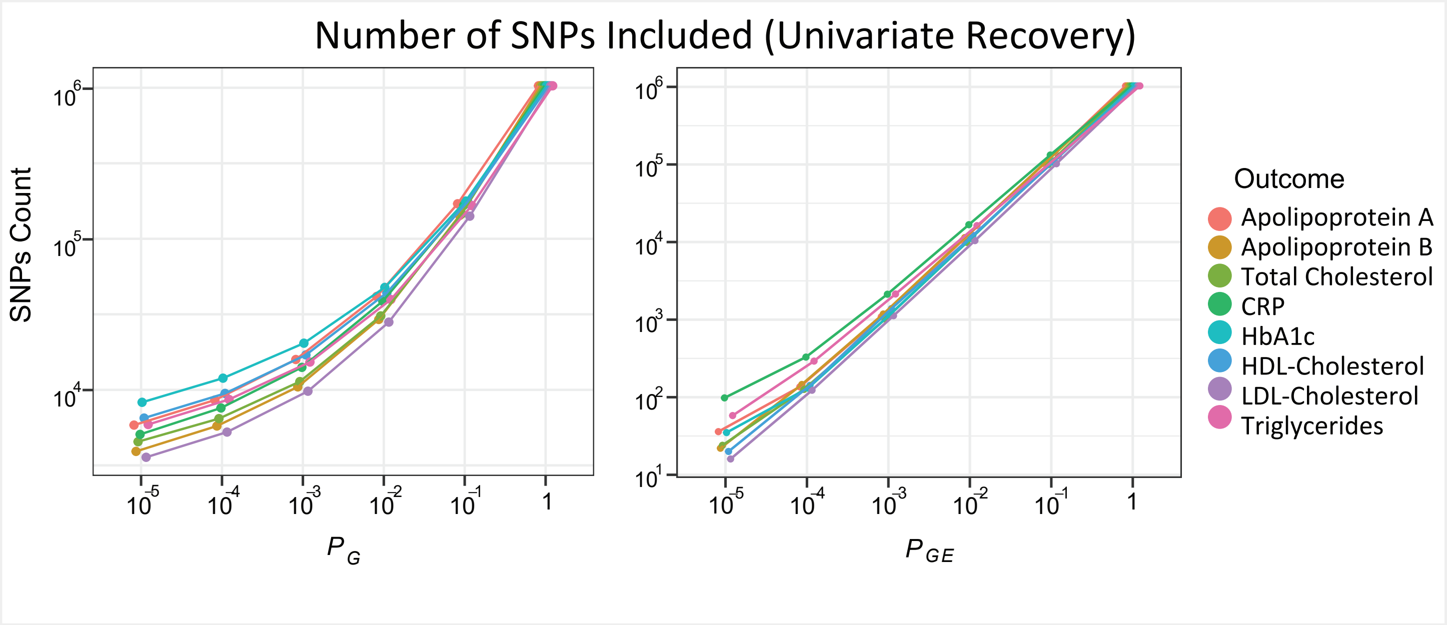

Supplementary Figure 5. Number of SNPs included by P_G and P_{GE} threshold for each outcome in the proportion of genetic and interaction variance recovered analysis. Source data are provided as a Source Data file.

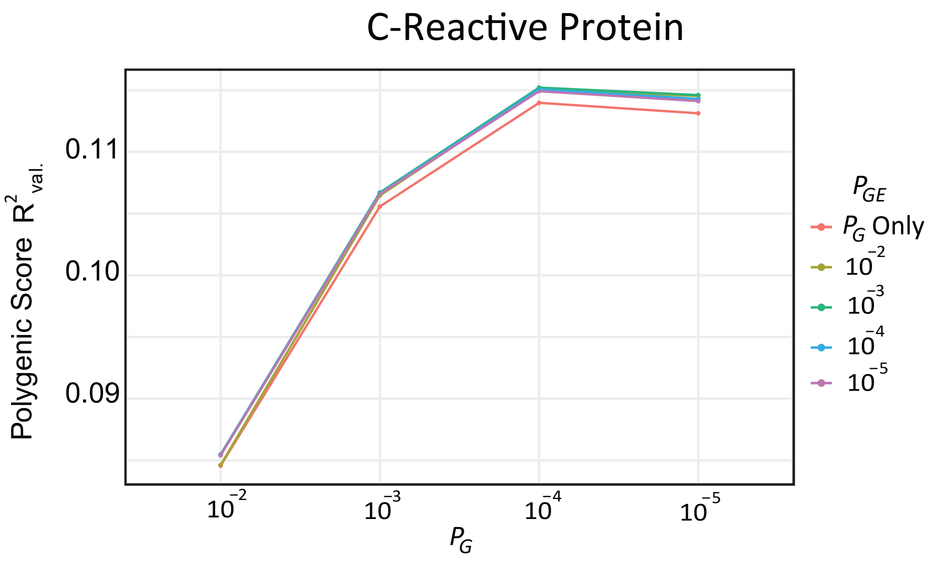

Supplementary Figure 6. Polygenic score prediction R^2 with and without incorporation of interaction effects for CRP. There are 20 different conditions based on discovery sample P_G and P_{GE} thresholds. The polygenic score R^2 was estimated in the validation sample based on discovery sample $\hat{\beta}_G$, $\hat{\beta}_{GE}$ values. Source data are provided as a Source Data file.
