# Supplementary material for: A versatile, fast and unbiased method for estimation of gene-by-environment interaction effects on biobank-scale datasets
Source: Nat Commun. 2023 Aug 25;14:5196. doi: 10.1038/s41467-023-40913-7 (PMC10457310; doi:10.1038/s41467-023-40913-7)
Supplement: Supplementary file 3 — Description of Additional Supplementary Files [file 41467_2023_40913_MOESM3_ESM.pdf]

File name: Supplementary Data 1

Description: Polygenic Scores with varying levels of GxE SNP inclusion for every outcome with confidence intervals. Statistical significance was measured using confidence intervals as per the MonsterLM method. Attached as supplementary data 1 file (.csv file).

File name: Supplementary Software 1

Description: MonsterLM_0.1.1. A compressed folder containing the Github code repository (MonsterLM software).
