# Supplementary figures and images for: A versatile, fast and unbiased method for estimation of gene-by-environment interaction effects on biobank-scale datasets
Source: Nat Commun. 2023 Aug 25;14:5196. doi: 10.1038/s41467-023-40913-7 (PMC10457310; doi:10.1038/s41467-023-40913-7)

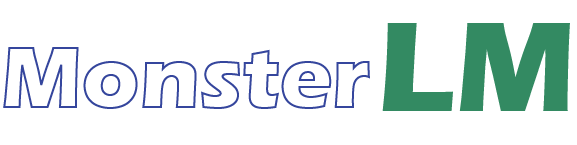

Supplement: Supplementary file 5 — Supplementary Software 1 [file 41467_2023_40913_MOESM5_ESM.zip › MonsterLM-0.1.1/MonsterLM_logo.0.2.png]

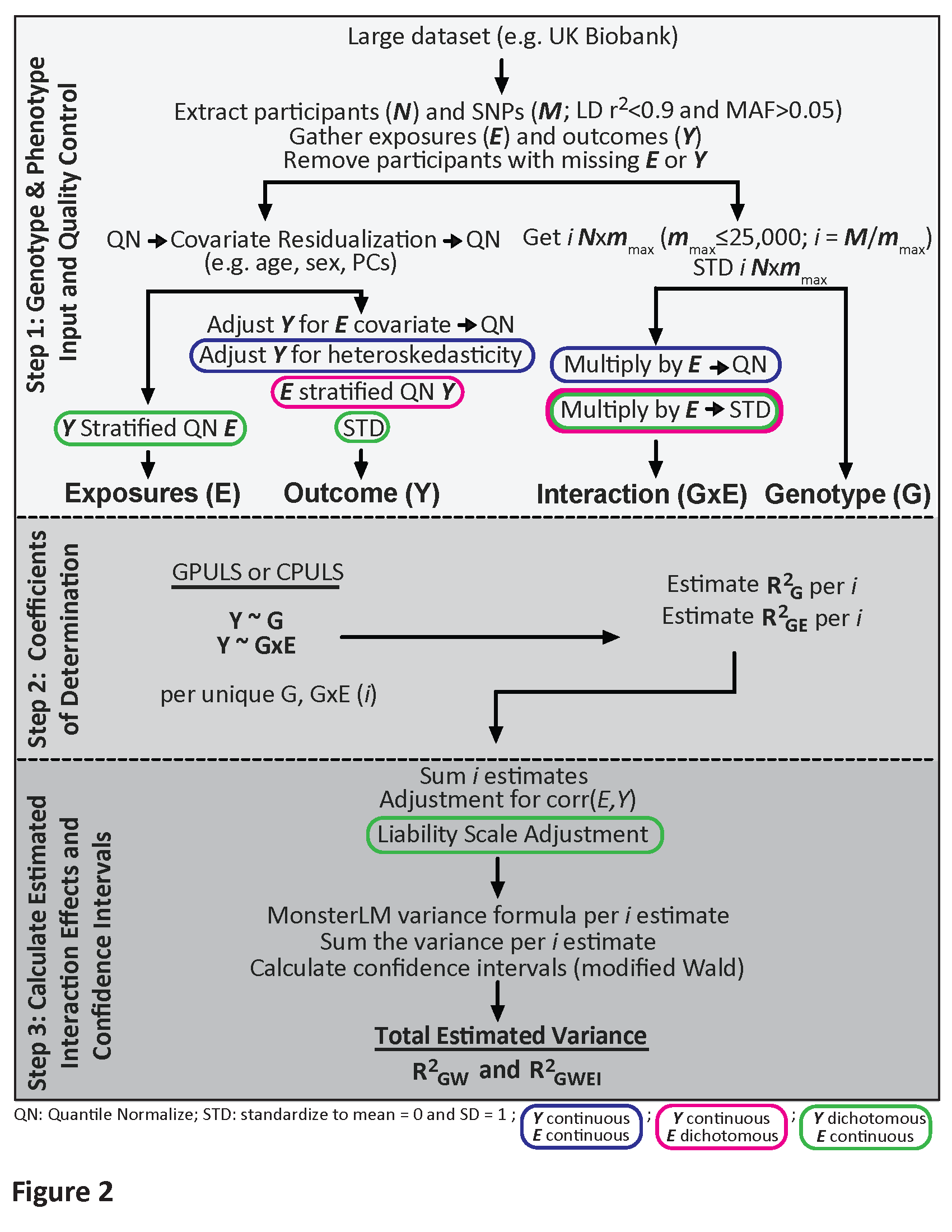

Supplement: Supplementary file 5 — Supplementary Software 1 [file 41467_2023_40913_MOESM5_ESM.zip › MonsterLM-0.1.1/method_overview_02.png]
