# Supplementary material for: A versatile, fast and unbiased method for estimation of gene-by-environment interaction effects on biobank-scale datasets
Source: Nat Commun. 2023 Aug 25;14:5196. doi: 10.1038/s41467-023-40913-7 (PMC10457310; doi:10.1038/s41467-023-40913-7)
Supplement: Supplementary file 6 — Reporting Summary [file 41467_2023_40913_MOESM6_ESM.pdf]

nature portfolio

Corresponding author(s): Dr. Guillaume Pare

Last updated by author(s): Jul 24, 2023

Reporting Summary

Nature Portfolio wishes to improve the reproducibility of the work that we publish. This form provides structure for consistency and transparency in reporting. For further information on Nature Portfolio policies, see our Editorial Policies and the Editorial Policy Checklist.

Statistics

For all statistical analyses, confirm that the following items are present in the figure legend, table legend, main text, or Methods section.

n/a	Confirmed	
☐	☒	The exact sample size (*n*) for each experimental group/condition, given as a discrete number and unit of measurement
☐	☒	A statement on whether measurements were taken from distinct samples or whether the same sample was measured repeatedly
☐	☒	The statistical test(s) used AND whether they are one- or two-sided<br>*Only common tests should be described solely by name; describe more complex techniques in the Methods section.*
☐	☒	A description of all covariates tested
☐	☒	A description of any assumptions or corrections, such as tests of normality and adjustment for multiple comparisons
☐	☒	A full description of the statistical parameters including central tendency (e.g. means) or other basic estimates (e.g. regression coefficient) AND variation (e.g. standard deviation) or associated estimates of uncertainty (e.g. confidence intervals)
☐	☒	For null hypothesis testing, the test statistic (e.g. *F*, *t*, *r*) with confidence intervals, effect sizes, degrees of freedom and *P* value noted<br>*Give P values as exact values whenever suitable.*
☒	☐	For Bayesian analysis, information on the choice of priors and Markov chain Monte Carlo settings
☒	☐	For hierarchical and complex designs, identification of the appropriate level for tests and full reporting of outcomes
☐	☒	Estimates of effect sizes (e.g. Cohen's *d*, Pearson's *r*), indicating how they were calculated

Our web collection on statistics for biologists contains articles on many of the points above.

Software and code

Policy information about availability of computer code

Data collection	Not applicable.
Data analysis	MonsterLM can be run on all major platforms (e.g. GNU/Linux, macOS, Windows). For biobank-scale analyses, recommended hardware requirements are a unix-like virtual environment supporting a minimum of 250 GB RAM space for in-memory operations. System GPUs are optional and can be used to speed up matrix inversion. Software requirements include the program dependencies: BASH (≥ 5.0), R (≥ 3.6.3), and GPULS (optional). Essential R dependencies include the packages: tidyverse, data.table, MBESS, and gsl. Individual-level biobank genotype and phenotype data processing is initially processed using PLINK version 1.9. Detailed documentation, code, and step-by-step implementation of the methodology in the main text is available on the managed GitHub repository: (https://github.com/GMELab/MonsterLM). A minting DOI version 0.1.1 is available on zenodo: (LINK).

For manuscripts utilizing custom algorithms or software that are central to the research but not yet described in published literature, software must be made available to editors and reviewers. We strongly encourage code deposition in a community repository (e.g. GitHub). See the Nature Portfolio guidelines for submitting code & software for further information.

Data

Policy information about availability of data

All manuscripts must include a data availability statement. This statement should provide the following information, where applicable:

- Accession codes, unique identifiers, or web links for publicly available datasets
- A description of any restrictions on data availability
- For clinical datasets or third party data, please ensure that the statement adheres to our policy

Individual genetic and phenotypic data were obtained from the UK Biobank (http://www.ukbiobank.ac.uk/), under application #15255. The UK Biobank study received approval from the National Health Service National Research Ethics Service North West. Access to the UK Biobank individual-level data is not publicly available and must be obtained via an application (https://www.ukbiobank.ac.uk/register-apply/). This paper includes the source data for all main and supplemental figures.

Research involving human participants, their data, or biological material

Policy information about studies with human participants or human data. See also policy information about sex, gender (identity/presentation), and sexual orientation and race, ethnicity and racism.

Reporting on sex and gender	The terms sex (biological attribute) and gender (social and cultural circumstances) are used appropriately throughout the manuscript. 54% of the cohort were females and 46% were males as defined by genetic sex.
Reporting on race, ethnicity, or other socially relevant groupings	Unrelated, white British individuals were chosen for the basis of this study as this was the largest single-ancestry cohort available in the UK Biobank.
Population characteristics	Cohort population characteristics are described in the "UK Biobank" section of the methods (lines 128 - 138): "The UK Biobank is a large population-based study which includes over 500,000 participants living in the United Kingdom22,23. Men and women aged 40–69 years were recruited between 2006 and 2010, and extensive phenotypic and genotypic data were collected. Quality control of genotype data was applied for individual and SNP inclusion using PLINK version 1.9. We selected 325,989 unrelated British individuals (the largest unrelated cohort; 54% female and 46% male) from the UK Biobank with both genotype and trait data for inclusion in the analysis. An unrelated set of individuals were chosen to reduce genomic prediction inaccuracies. Individual exclusion criteria included: (1) non-white British ancestry, (2) high ancestry-specific heterozygosity, (3) high genotype missingness (>0.05), (3) mismatching genetic ancestry, (4) sex chromosome aneuploidy, (5) mismatching gender sex and genetic sex, and (6) consent withdrawal at the time of analysis."
Recruitment	UK Biobank recruitment was conducted through a longitudinal study design (https://www.ukbiobank.ac.uk/). No local recruitment of individuals was conducted.
Ethics oversight	UK Biobank has approval from the North West Multi-centre Research Ethics Committee (MREC) as a Research Tissue Bank (RTB) approval. Our research group has access to the UK Biobank under the application #15255.

Note that full information on the approval of the study protocol must also be provided in the manuscript.

Field-specific reporting

Please select the one below that is the best fit for your research. If you are not sure, read the appropriate sections before making your selection.

☒ Life sciences ☐ Behavioural & social sciences ☐ Ecological, evolutionary & environmental sciences

For a reference copy of the document with all sections, see nature.com/documents/nr-reporting-summary-flat.pdf

Life sciences study design

All studies must disclose on these points even when the disclosure is negative.

Sample size	Sample sizes were determined by the maximum size of a single-ancestry cohort available in the UKB with the following exclusion criteria (manuscript lines 132 - 139): "We selected 325,989 unrelated British individuals (the largest unrelated cohort; 54% female and 46% male) from the UK Biobank with both genotype and trait data for inclusion in the analysis. An unrelated set of individuals were chosen to reduce genomic prediction inaccuracies. Individual exclusion criteria included: (1) non-white British ancestry, (2) high ancestry-specific heterozygosity, (3) high genotype missingness (>0.05), (3) mismatching genetic ancestry, (4) sex chromosome aneuploidy, (5) mismatching gender sex and genetic sex, and (6) consent withdrawal at the time of analysis."
Data exclusions	Individual sample size exclusion criteria included: "(1) non-white British ancestry, (2) high ancestry-specific heterozygosity, (3) high genotype missingness (>0.05), (3) mismatching genetic ancestry, (4) sex chromosome aneuploidy, (5) mismatching gender sex and genetic sex, and (6) consent withdrawal at the time of analysis." SNP exclusion criteria included: (1) Hardy-Weinberg disequilibrium (P<1×10−10), (2) highly correlated SNPs with an LD r2 > 0.9, (3) SNPs with a MAF < 0.05, (4) SNPs with low imputation quality (INFO score < 0.30), (5) call rate < 0.95, and (6) ambiguous or duplicated SNPs."
Replication	Genome-wide simulation analyses extensively tested for estimate reproducibility for 16 scenarios with 10 replicates or simulations per

Replication	scenario. Furthermore, follow-up analyses described in the methods that utilize discovery and validation sets with significant results offer replicated findings to the real data analyses.
Randomization	Not applicable. Our study is an observational study design. For follow-up analyses utilizing a discovery and validation, individuals were randomly allocated by selecting 80% to be used in the discovery cohort and 20% to be used in the validation cohort.
Blinding	Not applicable. Our study did not use any sort of intervention that would require blinding.

Reporting for specific materials, systems and methods

We require information from authors about some types of materials, experimental systems and methods used in many studies. Here, indicate whether each material, system or method listed is relevant to your study. If you are not sure if a list item applies to your research, read the appropriate section before selecting a response.

Materials & experimental systems

n/a	Involved in the study
☒	☐ Antibodies
☒	☐ Eukaryotic cell lines
☒	☐ Palaeontology and archaeology
☒	☐ Animals and other organisms
☒	☐ Clinical data
☒	☐ Dual use research of concern
☒	☐ Plants

Methods

n/a	Involved in the study
☒	☐ ChIP-seq
☒	☐ Flow cytometry
☒	☐ MRI-based neuroimaging
